# Serum levels of miR-320 family members are associated with clinical parameters and diagnosis in prostate cancer patients

**DOI:** 10.18632/oncotarget.23781

**Published:** 2017-12-30

**Authors:** Verena Lieb, Katrin Weigelt, Lena Scheinost, Kersten Fischer, Thomas Greither, Marios Marcou, Gerit Theil, Helmut Klocker, Hans-Juergen Holzhausen, Xin Lai, Julio Vera, Arif B. Ekici, Wolfgang Horninger, Paolo Fornara, Bernd Wullich, Helge Taubert, Sven Wach

**Affiliations:** ^1^ Department of Urology and Pediatric Urology, Universitätsklinikum Erlangen, Friedrich-Alexander-Universität Erlangen-Nürnberg, Erlangen, Germany; ^2^ Department of Urology, Martin-Luther-University Halle-Wittenberg, Halle, Germany; ^3^ Center for Reproductive Medicine and Andrology, Martin-Luther-University Halle-Wittenberg, Halle, Germany; ^4^ Department of Urology, Medical University Innsbruck, Innsbruck, Austria; ^5^ Institute of Pathology, Martin Luther University Halle-Wittenberg, Halle, Germany; ^6^ Laboratory of Systems Tumor Immunology, Department of Dermatology, Universitätsklinikum Erlangen, Friedrich-Alexander-Universität Erlangen-Nürnberg, Erlangen, Germany; ^7^ Institute of Human Genetics, Friedrich-Alexander-Universität Erlangen-Nürnberg, Erlangen, Germany

**Keywords:** miR-320 family, diagnosis, prostate cancer, PSA

## Abstract

We studied the association of the serum levels of the microRNA family members miR-320a/-b/-c with clinico-pathological data to assess their applicability as diagnostic biomarker in prostate cancer (PCa) patients. The levels of miR-320a/-b/-c in 3 groups were evaluated by qRT-PCR (145 patients with PCa, 31 patients with benign prostatic hyperplasia (BPH) and 19 healthy controls). The levels of the three family members of miR-320 were directly correlated within each group (*P* < 0.001), but they differed significantly among the three groups (*P* < 0.001). The serum levels of the miR-320 family members were significantly increased in older patients compared to younger patients (≤ 66 years vs. > 66 years, *P* ≤ 0.001). In addition, the levels of all three miR-320 family members were significantly different in patients with low tumor stage compared with those with high tumor stage (miR-320a: *P* = 0.034; miR-320b: *P* = 0.006; miR-320c: *P* = 0.007) and in patients with low serum PSA compared with those with high serum PSA (≤ 4 ng vs. > 4 ng; miR-320a: *P* = 0.003; miR-320b: *P* = 0.003; miR-320c: *P* = 0.006). The levels of these miRNAs were inversely correlated with serum PSA levels. Detection in the serum samples of PCa patients with or without PSA relapse revealed higher levels of miR-320a/-b/-c in the group without PSA relapse before/after radical prostatectomy than in that with PCa relapse.

In summary, the differences among the PCa/BPH/healthy control groups with respect to miR-320a/-b/-c levels in conjunction with higher levels in patients without a PSA relapse than in those with a relapse suggest the diagnostic potential of these miRNA-320 family members in PCa patients.

## INTRODUCTION

The microRNA miR-320 family is conserved, but only exists in vertebrates from Xenopus to humans. This miRNA family consists of five members: miR-320a, -b, -c, -d and -e (miR-320d/-e present only in primates and humans; Targetscan: http://www.targetscan.org/cgi-bin/targetscan/vert_71/mirna_families.cgi?db=vert_71&species=Human).

In the literature, the general term miR-320 is used most frequently, but when a sequence is given, it is predominantly that of miR-320a. miR-320 has been shown to regulate physiological processes such as cardiac survival (apoptosis) [1] and glucose-induced gene expression in diabetes [2], but most studies have investigated different tumor entities. miR-320 is downregulated in different tumors compared with normal/non-tumor tissue, such as in breast cancer [3–5], mesothelioma [6], liver cancer both in hepatic cholangiocarcinoma [7] and in hepatocellular carcinoma [8], lung cancer both in NSCLC [9] and SCLC [10], colon and colorectal cancer [11, 12], prostate cancer [13–15], oral cancer [16], and cervical cancer [17]. However, one report describes a 4-fold upregulation of miR-320 in neuroblastoma compared with normal retinal tissue [18]. The diagnostic utility of decreased miR-320 in the peripheral blood of glioblastoma patients compared with healthy probands has also been shown [19]. The downregulation of this miRNA was further associated with more aggressive behavior and/or poor prognosis in SCLC [10], colon cancer [11], breast cancer [3, 4], and cervical cancer [17]. Recently, an association between reduced miR-320a transcript levels in tumor tissue and a poor overall survival for prostate cancer patients was reported [15]. Taken together, this evidence not only shows the involvement of the miR-320 family in cancer development and progression, it also suggests the potential use of this microRNA family in clinical practice. However, an association of miR-320 family members with clinico-pathological data and their utility as diagnostic serum markers have not yet been studied in prostate cancer patients comprehensively.

## RESULTS

### General characteristics and expression levels of miR-320a, -b, and -c

The microRNA 320 family consists of five members (miR-320a-e). The members miR-320a, -b and -c showed detectable levels in the miRNA microarray but miR-320d and -e did not. Therefore, we excluded miR-320d and -e from further studies. The miR-320a gene is located on chromosome 8, whereas the miR-320b1/-2 genes are located on chromosome 1 and the miR-320c1/-2 genes are located on chromosome 18; however, the genes are expressed as one (miR-320a) or two transcripts (miR-320b1/-b2 and -c1/-c2). For all three miRNAs (miR-320a, -b, -c), the 3′ strand is predominantly expressed. The mature miRNAs have the following sequences (miRBase: http://www.mirbase.org/):

hsa-miR-320a-3p: 48 - aaaagcuggguugagagggcga - 69

hsa-miR-320b-3p: 39 - aaaagcuggguugagagggcaa - 60

hsa-miR-320c-3p: 50 - aaaagcuggguugagagggu - 69

(seed sequence is marked in red, and divergent nucleotides are marked in blue).

**Figure 1:**
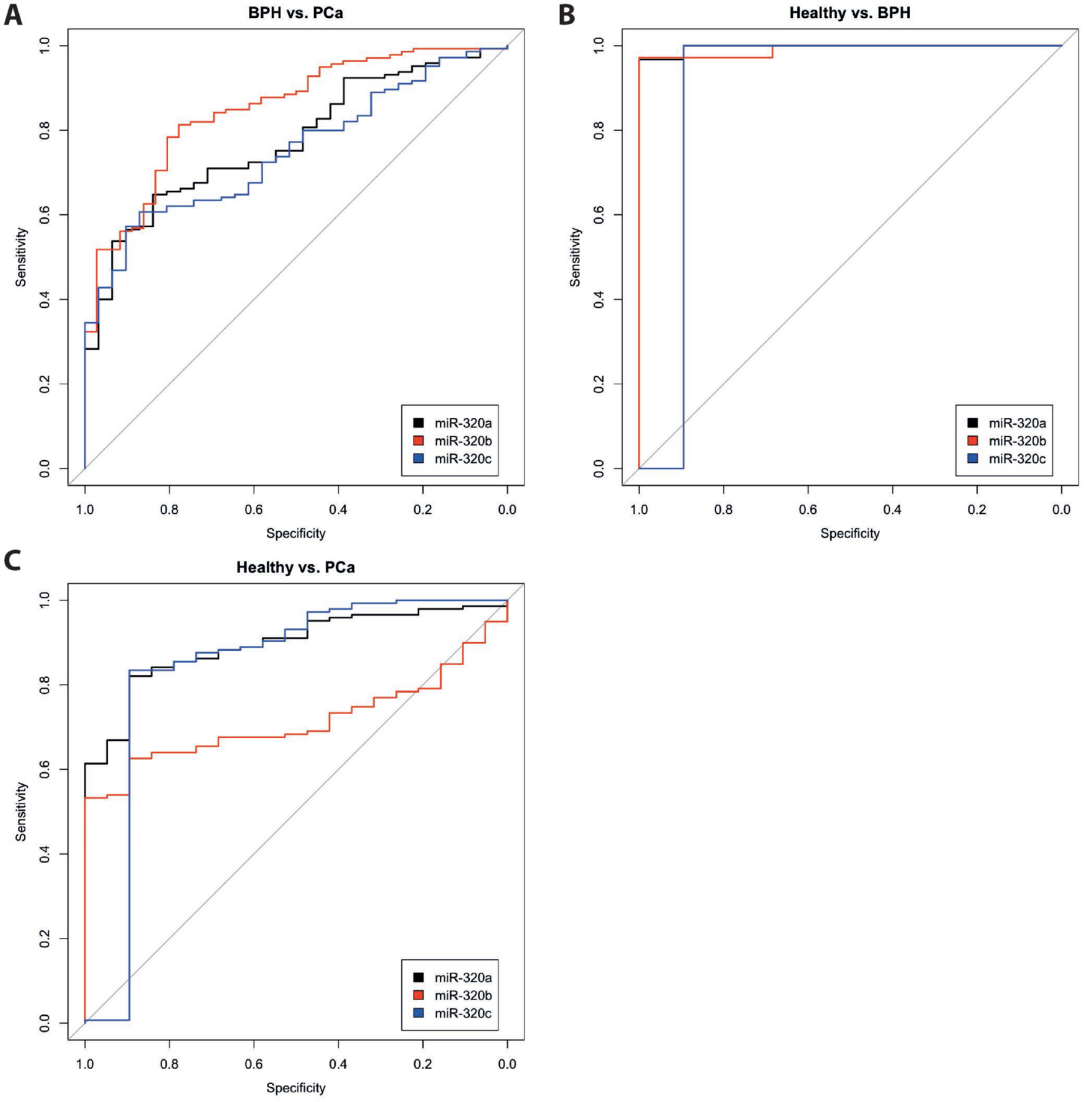
ROC analyses for miR-320 a, -b, and c in the serum of patients with PCa, patients with BPH and healthy controls The miRNA levels of miR-320a (black), miR-320b (red) and miR-320c (blue) were compared between the groups PCa vs. BPH (**A**), Healthy controls vs. BPH (**B**) and Healthy controls vs. PCa (**C**).

The expression levels (range, mean and median) of the three miRNAs miR-320a, -b and -c that were measured in the serum of 145 patients with PCa, 31 patients with BPH and 19 healthy donors are given in Table 1. The expression levels of all three miRNAs were highly correlated with each other in the serum samples of each of the three study groups (i.e., the PCa patients, the BPH patients and the healthy donors) (all *P <* 0.001; Spearman's rank correlation test; Table 2). However, the levels differed significantly between patients with PCa and those with BPH, and between both patient groups and the healthy controls (all *P* < 0.005; Mann-Whitney *U*-test and Kruskal Wallis test; Table 1; Supplementary Figure 1). Interestingly, the healthy control group showed the lowest median levels of these miRNAs in the serum, followed by the PCa group; finally, the BPH group showed the highest median expression levels of these miRNAs. ROC analyses revealed a distinction between patients with PCa and those with BPH (miR-320a: AUC of 0.775; miR-320b: AUC of 0.850 and miR-320c: AUC of 0.751), between patients with PCa and healthy controls (miR-320a: AUC of 0.894; miR-320b: AUC of 0.714 and miR-320c: AUC of 0.840) and between patients with BPH and healthy controls. However, the relatively low number of healthy controls must be considered (miR-320a: AUC of 0.997; miR-320b: AUC of 0.991 and miR-320c: AUC of 0.895; all with *P <* 0.005; Figure 1).

**Table 1:** Serum levels of miR-320a, -b, -c in PCa patients, BPH patients and healthy controls

	PCa	BPH	healthy controls	PCa vs. BPH	PCa vs. healthy controls	BPH vs. healthy controls
				P^1^	P^1^	P^1^
***miR-320a***				**<0.001**	**<0.001**	**<0.001**
***n***	145	31	19			
range	0.26–56.31	1.27–13.55	0.43–1.62			
mean	3.0666	4.9884	0.8566			
median	2.2388	3.7890	0.8542			
25% quartile	1.2005	3.0574	0.6515			
75% quartile	3.7975	5.9952	0.9836			
***miR-320b***				**<0.001**	**0.003**	**<0.001**
***n***	139	36	19			
range	0.42–219.79	1.97–41.36	0.82–2.75			
mean	5.7385	13.0849	1.7648			
median	2.9214	8.6052	1.8536			
25% quartile	1.4378	6.5050	1.3307			
75% quartile	5.4572	18.1321	1.8536			
***miR-320c***				**<0.001**	**<0.001**	**<0.001**
***n***	145	36	19			
range	0.35–167.90	1.17–13.62	0.25–19.91			
mean	3.6962	4.6928	2.4067			
median	2.0204	3.9124	0.5215			
25% quartile	0.8866	2.7534	0.3171			
75% quartile	3.6765	3.9124	0.5215			

^1^Mann-Whitney *U*-test; The Bonferroni-adjusted threshold for significance is set at α = 0.005.

**Table 2:** Bivariate correlations among the serum levels of miR-320a, -b, and -c in PCa patients, BPH patients and healthy controls

correlations					
PCa patients			miR-320a	miR-320b	miR-320c
Spearman-Rho	**miR_320a**	correlation coefficient	1.000	.893^**^	.937^**^
		Sig. (2-sided)		**.000**	**.000**
		N		138	145
	**miR_320b**	correlation coefficient		1.000	.876^**^
		Sig. (2-sided)			**.000**
		N			138
**BPH patients**			**miR-320a**	**miR-320b**	**miR-320c**
Spearman-Rho	**miR_320a**	correlation coefficient	1.000	.858^**^	.926^**^
		Sig. (2-sided)		**.000**	**.000**
		N		31	31
	**miR_320b**	correlation coefficient		1.000	.856^**^
		Sig. (2-sided)			**.000**
		N			31
**healthy controls**			**miR-320a**	**miR-320b**	**miR-320c**
**Spearman-Rho**	**miR_320a**	correlation coefficient	1.000	.818^**^	.930^**^
		Sig. (2-sided)		**.000**	**.000**
		N		19	19
	**miR_320b**	correlation coefficient		1.000	.786^**^
		Sig. (2-sided)			**.000**
		N			19

^**^The correlation is significant at the 0.001 level (2-sided).

### Correlation of miR-320 levels with clinico-pathological data

The serum level of all three miR-320 family members was not equally distributed when PCa patients were separated by median age (≤66 years vs. >66 years) according to their age at diagnosis (*P <* 0.001; Mann-Whitney *U*-test). After the levels of miR-320a, -b, and -c were separated by quartile, the lowest level (1st quartile) was found predominantly in the younger patient group while the highest level (4th quartile) was found predominantly in the elder patient group (miR-320a: *P* = 0.001; miR-320b and -c: *P <* 0.001; Fisher's exact test). However, after the classification of the BPH patients (≤70 years vs. >70 years) and the healthy controls (≤45 years vs. >45 years) according to median age, no differences were observed in the levels of all three miR-320 family members between the age groups.

In addition, the levels of all three miR-320 family members were not equally distributed between the two tumor stage groups (pT1 + pT2 vs. pT3 + pT4) (miR-320a: *P* = 0.034; miR-320b: *P* = 0.032 and miR-320c: *P* = 0.007; Mann-Whitney *U-*test). After the expression levels were stratified according to quartile, only miR-320a showed a significant association with a higher serum level (3^rd^ and 4th quartiles) and a higher tumor stage (*P* = 0.017). However, miR-320b and -c showed only a trend of significance for this association (*P* = 0.075 and *P* = 0.051; Fisher's exact test).

A different distribution of miR-320 family member levels was also seen in the two PSA groups (PSA level ≤ 4 ng vs. PSA level > 4 ng) (miR-320a: *P* = 0.003; miR-320b: *P* = 0.004 and miR-320c: *P* = 0.006; Mann-Whitney *U*-test). Interestingly, we found an indirect association between the serum levels of miR-320a/-b/-c and the serum levels of PSA. That is, low miR-320 levels (1st and 2nd quartiles) were associated with higher PSA levels and high miR-320 levels (3rd and 4th quartiles) were associated with lower PSA levels (miR-320a: *P* = 0.003; miR-320b: *P* = 0.006; and miR-320c: *P <* 0.001; Fisher's exact test).

### Early detection of PCa recurrence

**Figure 2:**
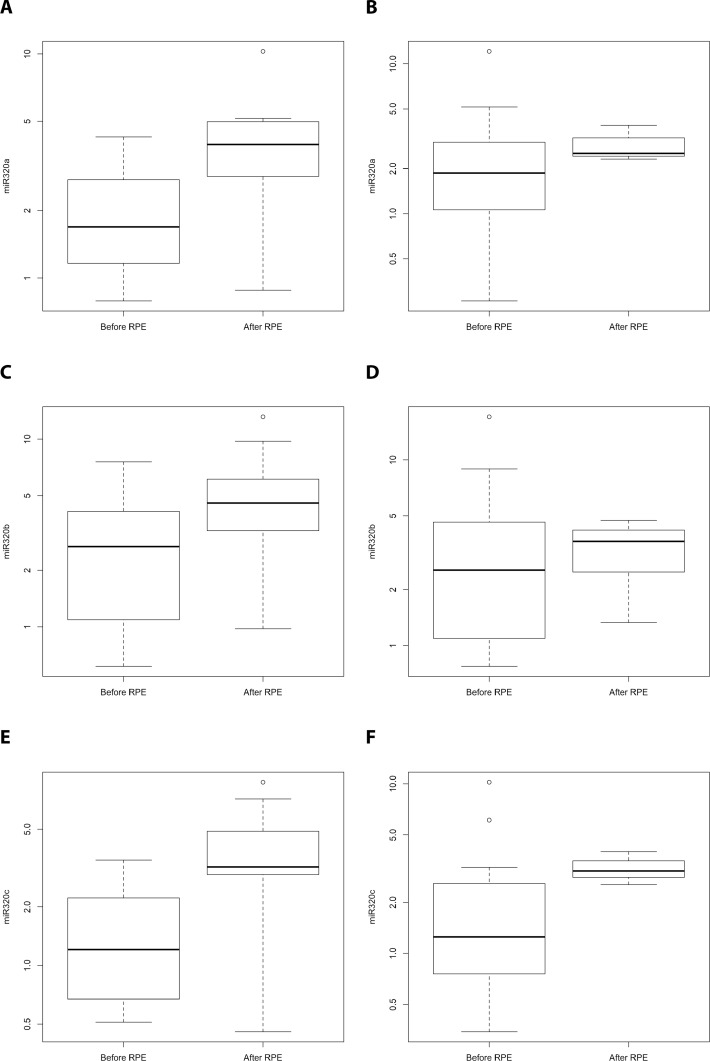
Comparison of miR-320a,-b and -c serum levels for PCa patients without/with PSA relapse before and after radical prostatectomy (Halle cohort) A comparison of miR-320a, -b and -c serum levels for PCa patients without (*N* = 36; **A**, **C**, **E**) and with PSA relapse (*N* = 41; **B**, **D**, **F**) before and after radical prostatectomy. There was a significant increase in miR-320a, -b and -c levels between samples before RPE and after RPE for the patients without PSA relapse (miR-320a: *P* = 0.019; miR-320b: *P* = 0.037 and miR-320c: *P* = 0.017) but not for the patients with PSA relapse (mir-320a: *P* = 0.177; miR-320b: *P* = 0.648; miR-320c: *P* = 0.089; all Mann Whitney *U*-test). Serum samples before and after the RPE originate from different PCa patients.

**Figure 3:**
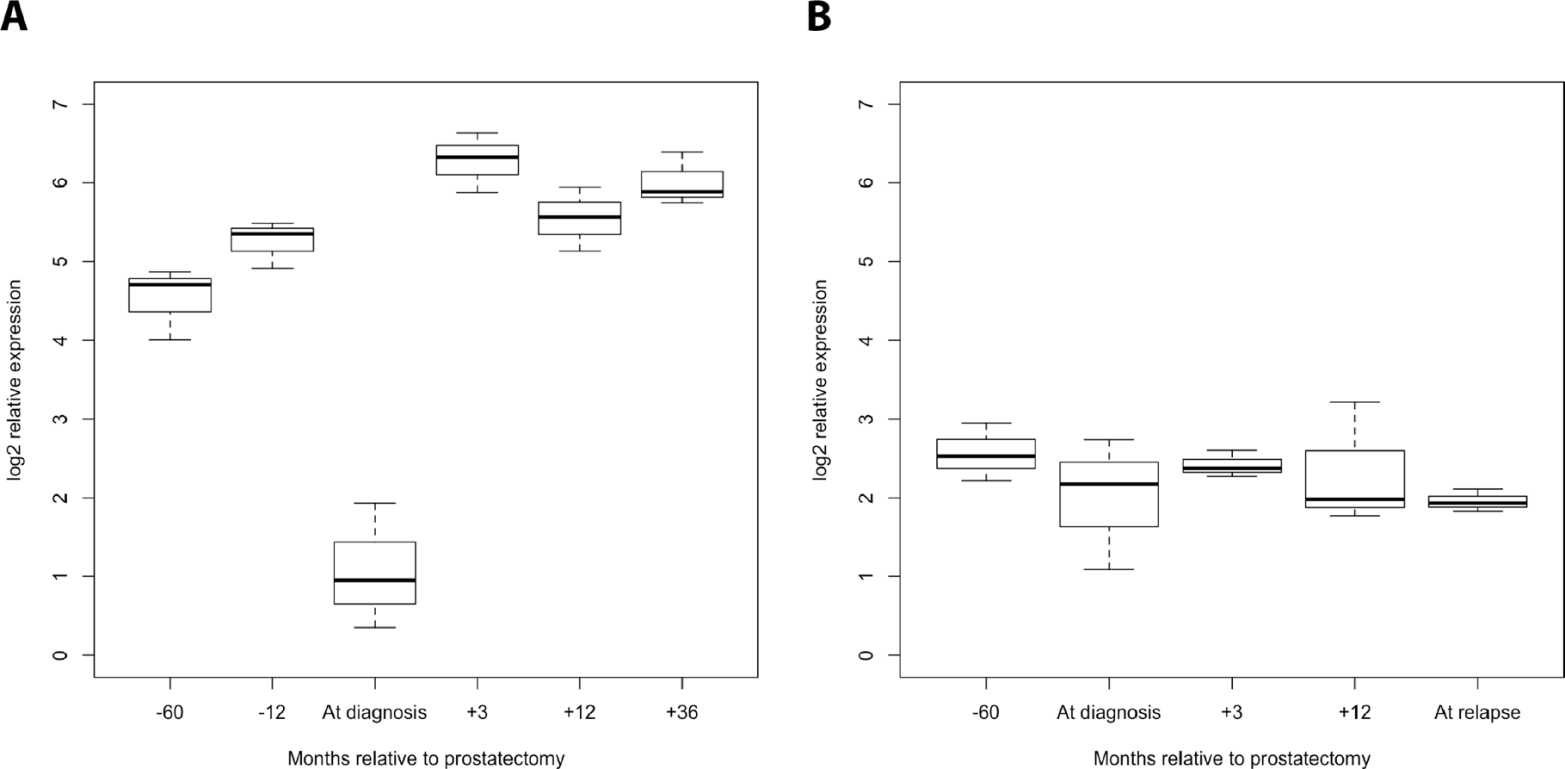
Progression profile of the levels of the miR-320 family members in the serum of PCa patients with/without PSA relapse (Innsbruck cohort) Serum samples (each pooled from 5 or 4 patients) obtained 5 years and one year before PCa diagnosis, at diagnosis, and 3 months, 1 year and 3 years after diagnosis were analyzed for the levels of miR-320a, -b, and -c in PCa patients without (**A**) and with PSA relapse (**B**). A strong decrease in all three miR-320 family members at the time of diagnosis was observed in PCa patients without PSA relapse but not in those with PSA relapse.

**Figure 4:**
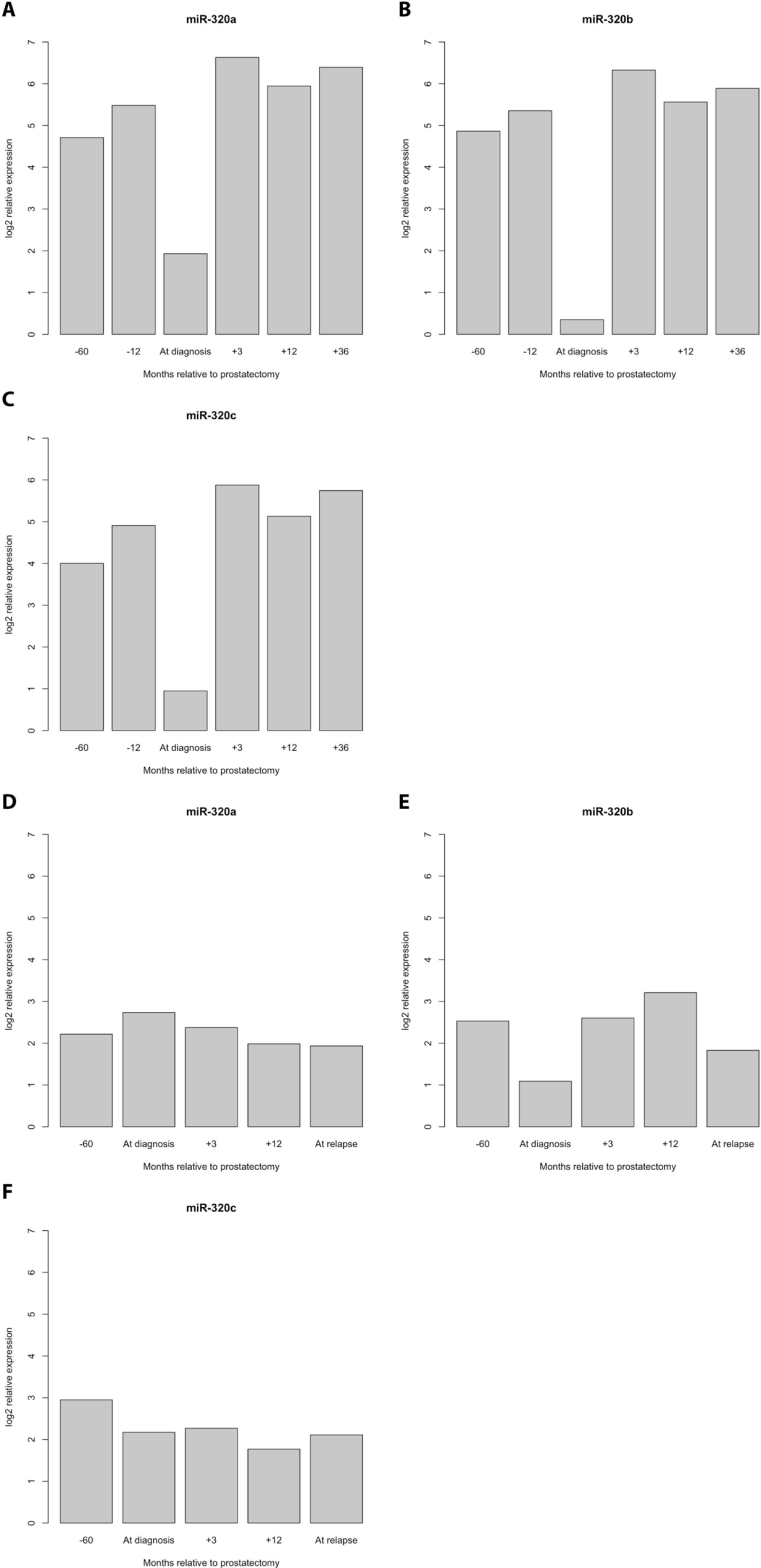
Detailed progression profile of the levels of the miR-320 family members for PCa patients without and with PSA relapse PCa patients without PSA relapse (**A**–**C**) All three miRNAs showed relatively high levels at 5 years and at 1 year before the diagnosis of PCa but a strong decrease at the time of PCa diagnosis. Remarkably, the previous high levels of miRNA-320 a, -b and -c were already reached three months after diagnosis and were maintained in PCa patients without PSA relapse. PCa patients with PSA relapse (**D**–**F**).

The detection of PCa recurrence (biochemical recurrence) is based on an increase in PSA levels (PSA relapse). Our finding of an indirect correlation between PSA expression and miR-320a, -b, and -c expression poses the question of if and how the serum levels of miR-320 family members might be associated with PCa recurrence. At first we investigated the PCa Halle cohort; for 77 patients (36 without and 41 with PSA relapse) the PSA relapse status was known. Comparison of miR-320a,-b and -c serum levels before and after radical prostatectomy (RPE) in the two PCa patient groups (without/with PSA relapse) revealed a significant increase in miR-320a, -b and -c levels between samples before and after RPE for the patients without PSA relapse (miR-320a: *P* = 0.019; miR-320b: *P* = 0.037 and miR-320c: *P* = 0.017) but not for the patients with PSA relapse (mir-320a: *P* = 0.177; miR-320b: *P* = 0.648; miR-320c: *P* = 0.089; all Mann Whitney *U*-test; Figure 2). Next, at using a microRNA microarray we studied serum samples from two patient groups (i.e., with and without PSA recurrence; *N* = 4 and 5, respectively) from the PCa Innsbruck cohort. In the group of PCa patients without recurrence, relatively high miR-320a/-b/-c levels were measured in serum samples obtained 5 years and 1 year before prostate cancer diagnosis. The levels were decreased in the serum collected at the time of diagnosis. Three months after RPE surgery and beyond, high miR-320a/-b/-c levels similar to those observed prior to surgery were again observed in the serum (Figures 3, 4). In the group of PCa patients with relapse, miRNA microarray analysis showed comparably lower levels of miR-320a/-b/-c in addition to a decrease in miR-320b (but not in miR-320a/-c) at diagnosis (Figures 3, 4). After RPE, the miR-320b levels increased to levels that were present before RPE, but this increase was less than that observed in the group without PSA relapse.

### Model to detect PCa recurrence

To calculate sensitivity and specificity for prediction of PCa recurrence after radical prostatectomy, we developed a generalized linar logistic regression model with clinico-pathological and molecular parameters. At first we integrated clinico-morphological parameters known to affect PCa recurrence, i.e., PSA level, Gleason sum, tumor stage, age and resection margins (R0 vs. R1) in our base line model and obtained a sensitivity of 77.8% and a specificity of 75.0% to separate PCa without from those with recurrence. At including miR-320a,-b,-c levels in addition, a sensitivity of 82.3% and a specificity of 77.8% is calculated. In the literature the level of miR-141 in serum/plasma has been described as relevant to distinguish between PCa without and with biochemical recurrence [20, 21]. Therefore, we analysed miR-141 level in our Halle cohort and added them to the previous model (PSA, Gleason sum, tumor stage, age and resection margins and miR-320a,-b,-c). A sensitivity of 93.7% and a specificity of 90.0% to distinguish between cases without/with PCa recurrence were achieved, suggesting that a model including clinico-pathological and several miRNA levels has an improved accuracy in the prediction of patients with PCa recurrence.

### Association of miR-320a, -b, and -c levels with prognosis

**Figure 5:**
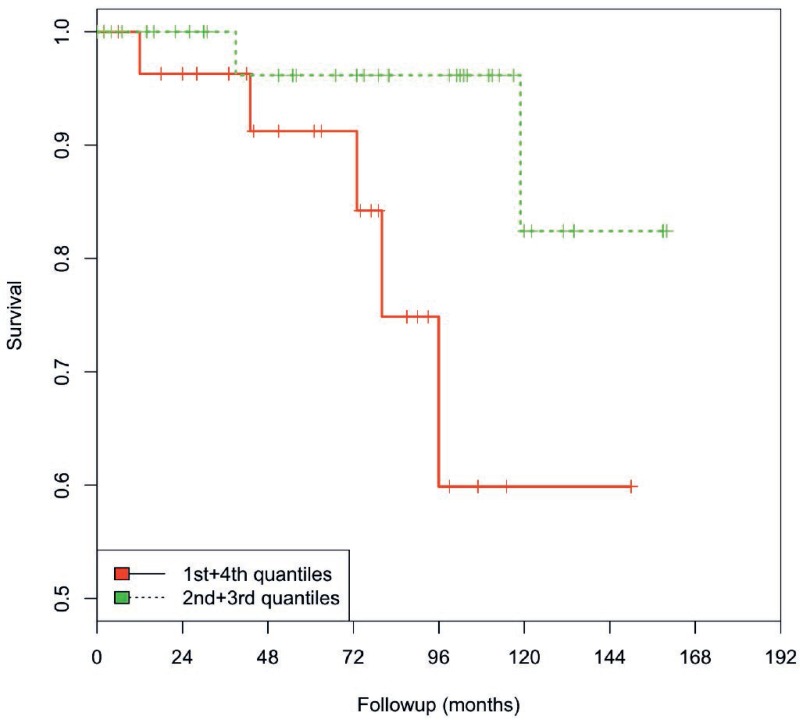
Kaplan-Meier analysis The levels of miR-320b in group 1 (1st and 4th quartiles/red line) compared with group 2 (2nd and 3rd quartiles/green line) were associated with OS in younger PCa patients (≤66 years). PCa patients with a low/high miR-320b level survived only 119 months compared with the patients with an intermediate miR-320b level (mean: 149 months; *P* = 0.034; log-rank test).

For the survival analysis, we separated the expression levels of miR-320a/-b/-c according to the quartiles. Interestingly, compared with intermediate expression levels (2nd and 3rd quartiles), the lowest and highest expression levels (1st and 4th quartiles) were associated with a poorer overall survival, although this difference was not statistically significant (data not shown). Therefore, we combined the 1st + 4th quartiles (group 1) and the 2nd + 3rd quartiles (group 2). Considering the association of miR-320a/-b and -c levels with age, patients were also stratified into two age groups (≤66 years vs. >66 years). In terms of miR-320b expression, group 1 patients (1st + 4th quartiles) of the younger patient group (≤66 years) showed a significantly poorer OS than patients in group 2 (2nd + 3rd quartiles) (*P* = 0.034; log- rank test). Moreover, group 1 patients died 30 months earlier than group 2 patients (119 vs. 149 months, Kaplan-Meier analysis; Figure 5) and had a 4.2-fold increased risk of death (statistically insignificant *P* = 0.12; multivariate Cox's regression hazard analysis, adjusted for PSA level and pT). A comparable effect was observed as trend for miR-320a levels (*P* = 0.053, log-rank test), as patients in group 1 had a 2.8-fold increased risk of death (statistically insignificant, *P* = 0.14; multivariate Cox's regression hazard analysis, adjusted for PSA level and pT; data not shown). In contrast, no association was observed between the levels of these miRNAs and overall survival in the elder patient group.

### *In silico* prediction of miR-320a, -b, and -c target genes

**Figure 6:**
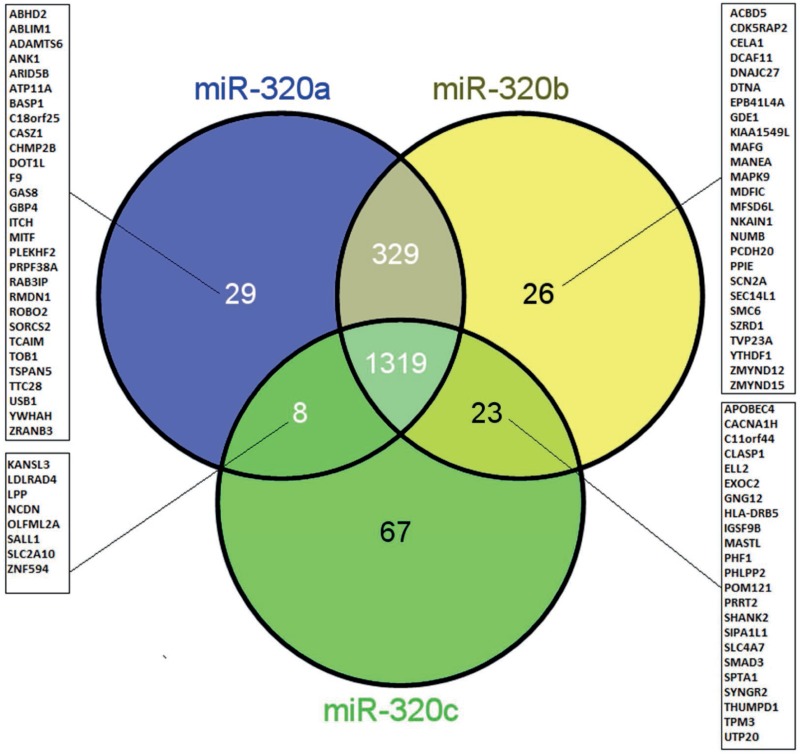
Venn diagram showing the overlap and singularity of predicted target genes of miR-320a, -b and -c The miRNA target genes were predicted using the database miRwalk2.0. A strong overlap of target genes (73%: 1319/1801) was observed for all three miRNAs.

Next, we were interested in the target genes of miR-320a, -b and -c as well as their overlap. First, we used the miRTarBase database to extract experimentally verified target genes of the three miRNAs, and based on the technologies used to verify miRNA-gene interactions, these targets were categorized into two groups, as follows: strong and weak confidence groups. Interactions in the former group were verified by experiments such as qPCR, western blot and reporter assays, while interactions in the latter group were verified by high-throughput experiments, such as microarray, RNA sequencing and pSILAC. When we consider only the methods with strong evidence, 20 target genes were reported for miR-320a, none for miR-320b and two for miR-320c (Supplementary Tables 1 and 2). Second, the database miRwalk2.0 was used to extract putative miRNA target genes that are predicted by 5 different algorithms (see Materials and Methods). In this way, 1685 genes were predicted for miR-320a, 1697 genes were predicted for miR-320b and 1417 genes were predicted for miR-320c. Interestingly, 73% (1319/1801; Figure 6) of the genes were predicted for all three miRNAs, 91.5% (1648/1801) were predicted for both miR-320a/-b, 74% (1327/1801) were predicted for both miR-320a/-c and 74.5% (1342/1801) were predicted for both miR-320b/-c. Only 1.6%, 1.4% and 3.7% were single target genes for miR-320a, -b and -c, respectively (Figure 6). To further elucidate the main pathways targeted by these miRNA target genes, we performed pathway enrichment analysis.

### Pathway enrichment analysis

For pathway enrichment analysis, three different programs were used: WIKI pathways, KEGG and REACTOME. We considered only pathways that were predicted to be significantly affected (adjusted *p*-value, *P* ≤ 0.05). Based on the results from the miRTarBase (high confidence target genes), several cancer pathways were identified using WIKI pathways and KEGG (Supplementary Table 1, marked in orange); the prostate cancer pathway was identified in KEGG (hsa05215; Supplementary Table 1, marked in yellow). Of note, the second most significantly enriched pathway extracted in KEGG with the high confidence target genes (second to the axon guidance pathway) was the PI3K-Akt signaling pathway (marked in ochre), which is one of the most affected pathways in prostate cancer [22]. Using putative miRNA targets obtained from miRWalk2.0, we also identified several cancer pathways in the three programs, including the prostate cancer pathway (hsa05215) in KEGG (Supplementary Table 1, marked in orange and in yellow).

## DISCUSSION

To study miR-320 family members and their association with clinico-pathological parameters as well as their diagnostic and prognostic utility, we analyzed serum samples obtained from liquid biopsies. The rationale for liquid biopsy is that tumors shed cells and/or genetic fragments, such as CTCs (circulating tumor cells) or cell-free nucleic acids (including miRNAs), into the circulation, which makes the blood/serum representative of PCa [23–26]. The levels of miR-320a/-b/-c in serum samples from patients with PCa, patients with benign prostatic hyperplasia (BPH) and healthy controls were significantly different, which suggests that all three miRNAs may serve as diagnostic biomarkers. In patients with PCa, a direct association was observed between an increased level of all three miR-320 family members and the age of these patients, but this was not the case for the patients with BPH or the healthy controls. Furthermore, the levels of all three miR-320 family members were directly associated with tumor stage but were inversely correlated with the level of PSA in the serum. Since a rising PSA level is a marker of PCa recurrence, we sought to determine if miR-320 levels are different between patients with/without PCa recurrence. PCa patients without a PSA relapse had significantly lower levels of all three miRNAs at the time of diagnosis compared with the pre-diagnosis or post-RPE time points, whereas PCa patients with a later PSA relapse showed a measurable decrease in the level of miR-320b at the time of diagnosis. In contrast, patients who underwent prostatectomy and who did not experience PSA relapse exhibited high levels of miR-320 a, b and -c after surgery, and PCa patients with relapse exhibited comparably lower levels of all three miRNAs. Altogether, miR-320a, -b and -c may serve as diagnostic biomarkers i) to distinguish PCa patients from those with BPH and from healthy controls, ii) to identify PCa patients early and iii) to identify those with a PSA relapse.

The low miR-320 levels measured in the serum at the time of PCa diagnosis are in agreement with the reported downregulation of miR-320 in prostate tumor tissue [14, 15]. With this in mind, we searched for miR-320 targets and possible functional consequences of miR-320 alterations. miR-320 (mostly miR-320a-3p) is considered to be a tumor suppressor miRNA, and several of its target genes/proteins that are involved in different physiological and tumor pathways (e.g., ß-catenin, Myc, STAT3) have been reported (Supplementary Tables 1 and 2).

The miRwalk program *in silico* also identified the androgen receptor (AR) as a direct target gene/protein of miR-320a, -b, and -c (Supplementary Table 1). This was recently confirmed by Sato and colleagues who identified the binding (seed) sequence of miR-320a (3′-GUCGAAA-5′) to AR, which is also present in miR-320b and -c; this suggests that all three family members regulate the expression of AR. In addition, the transcription factor SP1, which activates the transcription of AR [27], is also an *in silico* target of miR-320a, -b and -c (Supplementary Table 1). The downregulation of AR by miR-320a, -b and -c would result in decreased transcription of the AR target PSA, which would explain the inverse correlation of PSA and miR-320 levels.

Sato and colleagues also reported that a low miR-320a level was associated with poor OS [15]. After an examination of the correlation of miR-320a, -b, and -c levels with OS in this study, only a relationship between a low/high level (1st and 4th quartiles) of miR-320b, as opposed to intermediate levels (2nd and 3rd quartiles), and a poorer OS in younger PCa patients (≤66 years) was observed. The surprising finding that both a high and a low level are associated with a poorer prognosis suggests that both a reduction and an increase in miR-320b can support tumor development. It was proposed that miRNA-320b may compete with miRNA-320a, which would therefore up-regulate the target genes of miRNA-320a including β-catenin, Neuropilin-1 and Rac-1. These genes are all known as promoters of tumor proliferation, invasion and metastasis in colorectal cancer [28]; reviewed in Supplementary Tables 1 and 2. This remarkable mechanism exemplifies the control of tumor promotion pathways via homologous competition between miRNAs of the same family.

Since we observed an association only between the level of miR-320b and prognosis, we were interested in the commonalities and the differences in the miR-320 family members with respect to their proposed target genes. After the application of miRwalk, we found that 73% (1319/1801) of the genes predicted by at least five prediction programs were shared by miR-320a/-b and -c (Figure 6). However, miR-320b also has 26 predicted target genes that are not shared by the other two members (Figure 6). Of these, six genes (MANEA, MAPK9, NKAIN1, NUMB, SCL14L1, and SMC6) have been reported in PubMed to play a role in prostate cancer (https://www.ncbi.nlm.nih.gov/pubmed). MANEA has been described as an androgen responsive gene [29]. Expression of the MAPK9 gene is higher in metastatic prostate tumors than in primary prostate tumors [30]. Expression of the NKAIN1 gene is higher in the urinary sediment of PCa patients than in normal controls, and among PCa patients, NKAIN1 expression is higher in patients with Gleason scores ≥7 than in patients with Gleason scores ≤6, which suggests its utility as an early diagnostic marker of PCa [31]. NUMB (protein numb homologue) is a key regulator of cell fate that controls NOTCH and GLI, which play major roles in prostate cancer [32]. SCL14L1 expression has been associated with a high combined Gleason score, advanced tumor stage, and PSA progression, and therefore, may be used as a biomarker of progression in PCa [33]. Increased expression of the SMC6 gene after irradiation is associated with DNA damage and repair and has been reported in PCa patients [34]. However, until now, none of these miR-320b predicted target genes/proteins have been confirmed by high-evidence methods. Altogether, we suggest that combination of several miRNA levels (including miR-320 family members and miR-141) can help to improve the accuracy of future risk models for PCa recurrence. But of course our results should be checked in larger, independent and prospective patient cohorts.

In summary, we showed that the levels of miR-320a, -b and -c are associated with each other within the different groups of PCa patients, BPH patients and healthy controls but that the levels differ significantly among these groups. The serum levels of all three miR-320 family members are significantly different in patients with low-stage tumors compared with those with advanced-stage tumors (pT1 + pT2 vs. pT3 + pT4) and are directly correlated to pT and indirectly correlated to PSA serum levels. PCa patients without tumor recurrence (PSA relapse) have higher levels of miR-320a/-b/-c before and after radical prostatectomy compared with patients with PCa recurrence. In addition, miR-320b levels (lowest and highest quartiles) were found to be associated with poor OS in younger PCa patients (≤66 years). The prediction of miR-320a, -b, and -c target genes reveals a great overlap of potential target genes of all three miRNAs, and pathway enrichment analysis detected several cancer-related pathways. Altogether, the differences between the PCa/BPH/healthy control groups with respect to miR-320a/-b/-c levels in conjunction with higher levels in patients without a PCa relapse suggest the diagnostic utility of these miRNA-320 family members in PCa patients.

## MATERIALS AND METHODS

### Clinical samples

The miRNA levels of miR-320a, -b and c were analyzed in the serum of 145 non-selected patients with PCa (Halle cohort), 31 patients with BPH and 19 healthy controls. All of the patients were treated at the Department of Urology at Martin-Luther-University Halle-Wittenberg from 1995–2005. Blood samples were collected during routine diagnostic examinations. All patients provided written informed consent. To study the miR-320a, -b, and -c levels in PCa patients with or without PSA relapse, a second PCa cohort (Innsbruck cohort) from the PCa early detection program in Tyrol, Austria was analyzed [35]. The group of PCa patients with PSA relapse consisted of five patients while the group without PSA relapse consisted of four patients. Serum samples from both groups were assessed by microRNA microarrays to determine the levels of miR-320a, -b and -c. In the group of patients without recurrence, serum samples were obtained at 5 years and at 1 year before the diagnosis of PCa, at diagnosis, and at approximately 3 months, 1 year and 3 years after radical prostatectomy (RPE). In the group of patients with recurrence, serum samples were available at 5–6 years before diagnosis, at diagnosis, and at approximately 3 months and 1 year after RPE, and at relapse. This study was performed in compliance with the Declaration of Helsinki. The use of blood samples for research was approved by the Internal Review Boards of the Medical Faculty of the Martin-Luther-University Halle-Wittenberg and the Ethics Committee of the Medical University Innsbruck. The tumors were staged according to the Union for International Cancer Control system, and they were graded according to the Gleason score system. The relevant clinico-pathological parameters of the PCa patient cohorts are presented in Table 3A and 3B.

**Table 3A:** Clinico-pathological data of the PCa cancer patients from the Halle cohort

	PCa	BPH	healthy controls
***N***	145	31	19
**Age**			
range	44–91	55–89	40–57
mean	65.7	70.4	46.4
median	66.0	70.0	45.0
**Gleason sum**			
<7	40	n.a.	n.a.
=7	54	n.a.	n.a.
>7	41	n.a.	n.a.
unknown	10	n.a.	n.a.
**Tumor stage**			
T1/2	98	n.a.	n.a.
T3/4	44	n.a.	n.a.
unknown	3	n.a.	n.a.
**PSA**			
<4 ng	45	n.a.	n.a.
≥4 ng	100	n.a.	n.a.
**Overall survival**			
alive	115	31	19
dead	30	0	0
**Disease-specific survival**			
alive	135	31	19
dead	10	0	0

n.a.-not applicable.

**Table 3B:** Clinico-pathological data of the PCa patients from the Innsbruck cohort

PCa Patients	ID	age	PSA at biopsy (Diagnosis)	GS at biopsy	GS at RPE	pT	time between RPE and PSA relapse (in days)
Without progression							
	wo1	58	2.8	6	5	2b	n.a.
	wo2	63	6.0	7	8	2c	n.a.
	wo3	78	n.d.	6	6	2c	n.a.
	wo4	71	6.19	6	7	2c	n.a.
	wo5	68	2.96	6	7	2c	n.a.
With progression							
	w1	64	2.03	8	7	3a	841
	w2	66	4.96	7	7	3b	1263
	w3	63	9.0	5	7	2c	1114
	w4	61	1.97	7	9	2c	1653

n.d.-not determined, n.a.-not applicable.

### miRNA quantitative real-time PCR

Serum miRNAs were purified using a miRCURY RNA isolation kit for body fluids (Exiqon, Vedbaek, Denmark). For the miRNA analysis, isolated RNA corresponding to 25 μl of serum was reverse transcribed using a universal cDNA synthesis kit (Exiqon). Real-time PCR was performed with the StepOnePlus Real-Time PCR System (Life Technologies, Darmstadt, Germany) using sequence-specific primers for miR-320a/b/c and miR-141 (Exiqon). The PCR reactions were performed in triplicate in a final volume of 10 μl containing 1x SYBR green PCR Master Mix, 1x sequence-specific primer mix, and cDNA, which corresponded to a miRNA amount of 5 μl serum per real-time PCR reaction. The thermal cycling conditions were selected according to the manufacturer's recommendations. To quantify the levels of miR-320a/b/c in the serum, we used the relative quantification (ΔΔCt) method [36] and used miR-16-5p and miR425-5p as internal controls. All calculations were performed with the StepOne software V 2.0 (Life Technologies).

### miRNA differential expression analysis using microarrays

The miRNA microarray expression analyses were performed on GeneChip miRNA 4.0 microarrays (Affymetrix, Santa Clara, CA, USA) according to the manufacturer's instructions. The array contained sequence-specific probes for 2,578 human mature miRNAs and 2,025 human pre-miRNAs listed in miRBase v20.0 (http://www.mirbase.org). The resulting data were further analyzed with Partek Genomics Suite software v6.6 (Partek, St Louis, MO, USA). To identify miRNAs differentially expressed between the defined sample groups, ANOVA test statistics were applied.

### miRNA target genes

We used miRTarBase 2016 (release 6.0) to identify experimentally verified miRNA targets [37]. Those targets that were verified by reliable technologies (e.g., qPCR, western blot and reporter assays) were chosen for pathway enrichment analyses. The miRwalk2.0 database was used to identify the target genes of the identified deregulated miRNAs [38]. The putative miRNA target genes predicted by five independent algorithms, including miRwalk (v2.0), DIANA-microT (v4.0), MiRanda (released in 2010), RNA22 (v2) and Targetscan (v6.1), were extracted for the subsequent analyses. This allows a reduction in the number of false-positive results, which is a common issue in existing miRNA target prediction programs [39].

### Pathway enrichment analyses

We used the obtained miRNA target genes to perform pathway enrichment analyses. These gene lists were used as the inputs for the web-based platform Enrichr [40]. The outputs are lists of biological pathways/terms to which given miRNA target genes belong. The biological pathways/terms were derived from curated databases such as KEGG [41], WIKIPathways [42] and Reactome [43]. Fisher's exact test was employed to determine the significance of a biological pathway/term to the given gene lists. The pathways/terms with corrected *p*-values ≤ 0.05 (adjusted using the Benjamini-Hochberg procedure) were regarded as significant.

### Statistical analysis

According to the exact time point of blood collection, the PCa patients were classified into one of the following three groups: (1) patients who did not undergo radical prostatectomy and who were diagnosed with PCa based solely on biopsy specimens, (2) patients whose blood was collected before radical prostatectomy, and (3) patients whose blood was collected at least six months after radical prostatectomy in order to exclude post-surgery effects on the miR-320 levels. The distribution of miR-320a, -b, and -c did not differ among the three defined groups (data not shown), and therefore, we did not further distinguish among them in this study. Correlations between continuous variables of the biological markers were calculated by Spearman's rank correlation test (r_s_). For further statistical analysis, expression levels were separated into four groups according to the 25%, 50% and 75% percentiles (≤25%, >25% to ≤50%, >50% to ≤ 75% and >75%). The differences among the levels of miR-320a, -b, and -c in the serum of patients with PCa and BPH and healthy volunteers were estimated using the Mann-Whitney *U*-test and the Kruskal Wallis test. For survival analyses in patients with PCa, OS was defined as the time from diagnosis to death, which was used as the follow-up end point. Statistical analyses of the association between miR-320a, -b, and -c levels and prognosis were performed according to the Kaplan-Meier method (log-rank test) and multivariate Cox's proportional hazard regression models. The multivariate Cox's regression hazard model was adjusted to PSA level and tumor stage. All calculations were performed using the SPSS 20.0 statistical package (SPSS-Science, Chicago, IL, USA) and R Ver. 3.2.1 (R Foundation for Statistical Computing, Vienna, Austria. http://www.R-project.org/).

## SUPPLEMENTARY MATERIALS FIGURES AND TABLES







## Abbreviations

PCa: prostate cancer
PSA: prostate specific antigen
OS: overall survival
RPE: radical prostatectomy
BPH: benign prostatic hyperplasia
