# Supplementary material for: Serum levels of miR-320 family members are associated with clinical parameters and diagnosis in prostate cancer patients
Source: Oncotarget. 2017 Dec 30;9(12):10402–16. doi: 10.18632/oncotarget.23781 (PMC5828216; doi:10.18632/oncotarget.23781)
Supplement: Supplementary file 1 [file oncotarget-09-10402-s001.pdf]

Serum levels of miR-320 family members are associated with clinical parameters and diagnosis in prostate cancer patients

SUPPLEMENTARY MATERIALS

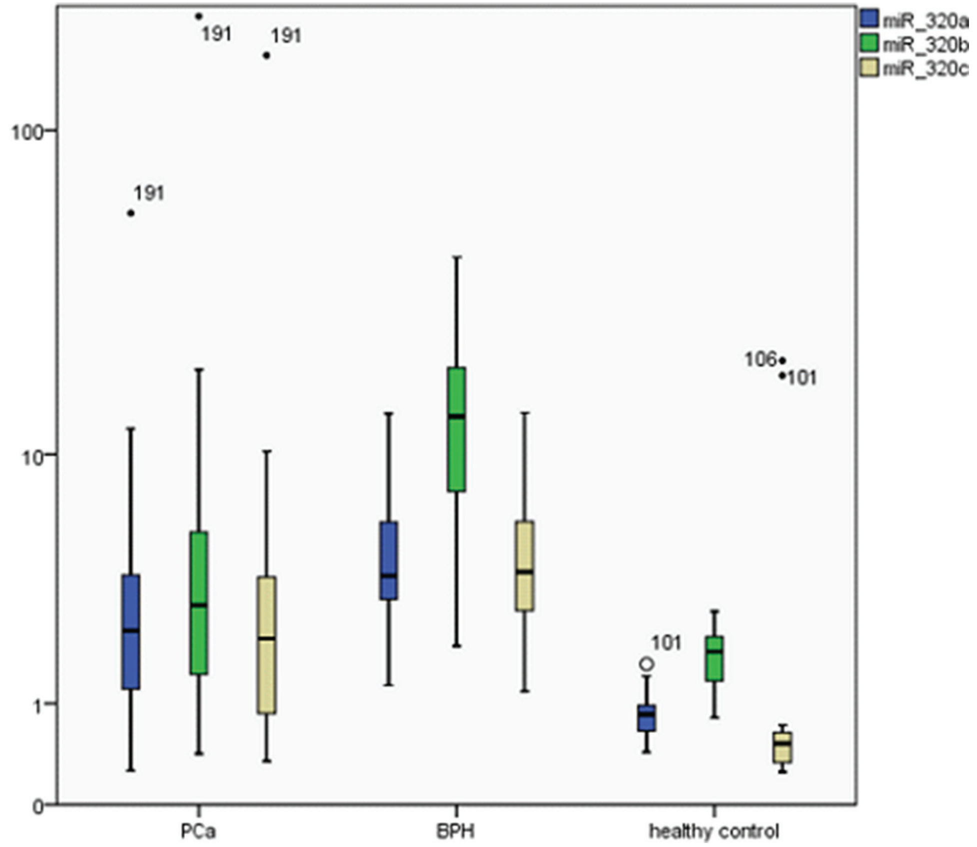

Supplementary Figure 1: Box plot: Expression levels of miR-320a,-b and –c in the three groups PCa, BPH and healthy control.

Supplementary Table 1: Target analysis for mir-320a/-b/-c and pathway enrichment analysis. See Supplementary_Table_1

Supplementary Table 2: Validated target genes/proteins of the miR-320 family members. See Supplementary_Table_2
