# Supplementary material for: Serum levels of miR-320 family members are associated with clinical parameters and diagnosis in prostate cancer patients
Source: Oncotarget. 2017 Dec 30;9(12):10402–16. doi: 10.18632/oncotarget.23781 (PMC5828216; doi:10.18632/oncotarget.23781)
Supplement: Supplementary file 3 [file oncotarget-09-10402-s003.docx]

**Supplementary Table 2 Validated target genes/proteins of the miR-320 family members**

| **gene** | **gene** | **function** | **reference** |
| --- | --- | --- | --- |
| **human miR-320a** |  |  |  |
| POLR3D (promoter) | polymerase III, RNA subunit D | cell cycle regulation | Ref.1 |
| MCL-1 | myeloid cell leukemia-1 | antiapoptotic protein | Refs. 2,3 |
| TFRC/CD71 | transferrin receptor 1 | cellular iron uptake | Ref. 4 |
| HSPB6/HSP20 | heat shock protein 20 | chaperone, protective in stressful situations | Refs. 5,6 |
| AQP1 | aquaporin 1 | water channel protein, angiogenesis & tumor spread | Refs. 7,8 |
| AQP4 | aquaporin 4 | water channel protein | Ref. 8 |
| NRP1 | neuropilin 1 | co-receptor of vascular epithelial growth factor | Ref. 9 |
| MAPK1/ERK2 | mitogen-activated protein kinase 1/extracellular signal regulated kinase 2 | eukaryotic signal transduction | Ref. 10 |
| GNAI1 | guanine nucleotide-binding protein, α-inhibiting activity polypeptide 1 | G-protein, signal transduction | Ref. 11 |
| COX2 (promoter) | cyclooxygenase 2 | regulation of proinflammatory cytokines | Ref. 12 |
| CTNNB* | β-catenin | Wnt/ß-catenin signaling pathway | Ref. 13 |
| IGFR1 | insulin growth factor 1 receptor | growth hormone receptor, cell cycle progression | Ref. 14 |
| BANP/SMAR1 | BTG3-associated nuclear protein | erythroid differentiation | Ref. 15 |
| FASN | fatty acid synthase | synthesis of long-chain saturated fatty acids | Ref. 16 |
| NFATC3 | nuclear factor of activated T-cells, cytoplasmic calcineurin-dependent c3 | transcription factor, activation of cytokine genes | Ref. 17 |
| TRPC5 | transient receptor potential cation channel, subfamily C, member 5 | potential calcium channel protein | Ref. 17 |
| ARF1 | ADP ribosylation factor1 | guanine nucleotide-binding protein, vesicular transport | Ref. 18 |
| BMI1 | leukemia viral BMI1 oncogene, mouse, homolog of BMI1 | Regulates proliferative activity of normal stem & progenitor cells | Ref. 19 |
| ITGB3/CD61 | integrin ß3 | platelet aggregation, receptor for fibrinogen | Ref. 20 |
| RAC1 | ras-related C3 botilinim toxin substrate 1 | regulation of actin filaments at the plasma membrane | Ref. 21 |
| E2F1 | E2F transcription factor 1 | transcription factor | Ref. 22 |
| FOXM1* | forkhead box M1 | control of cell proliferation | Ref. 23 |
| LAMP1 | lysosomal-associated membrane protein 1 | lysosomal exocytosis | Ref. 24 |
| MYC | v-myc avian myelocytomatosis viral oncogene homolog | oncogene, DNA-binding and transcription factor | Ref. 25 |
| CRKL | v-CRK avian sarcoma virus CT10 oncogene homolog-like | oncogene, tyrosine kinase | Ref. 26 |
| STAT3 | signal transducer and activator of transcription 3 | oncogene, transcription factor | Ref. 27 |
|  |  |  |  |
| **human miR-320c** |  |  |  |
| GNAI1 | guanine nucleotide-binding protein, α-inhibiting activity polypeptide 1 | G-protein, signal transducing | Ref. 11 |
| SMARCC1 | SWI/SNF-related, matrix-associated, actin-dependent regulator of chromatin, | chromatin remodelling | Ref. 28 |
|  | subfamily C, member 1 |  |  |
| SOX4 | SRY-box 4 | lymphocyte-specific transcriptional activator | Ref. 29 |
| FOXM1* | forkhead box M1 | control of cell proliferation | Ref. 29 |
| FOXQ1 | forkhead box Q1 | cell cycle regulation | Ref. 29 |
| **murine miR-320** |  |  |  |
| IGF-1 | insulin-like growth factor-1 | mediates growth-promoting effects of growth hormones | Ref. 30 |
| ETS2 | erythroblastosis virus E26 oncogene homolog 2 | transcription factor, endothelial cell survival | Ref. 31 |
| MMP9 | matrix metalloproteinase 9 | extracellular matrix composition | Ref. 31 |
| EMELIN2 | elastin microfibril interfacer 2 | extracellular matrix composition | Ref. 31 |

*binding sequence present in miR-320a, -b, and -c

This table is based on experimentally verified target genes in the miRTarBase database and in a PubMed search for validated target genes of miR-320 family members.

References

1. Kim DH, Saetrom P, Snove O, Jr., Rossi JJ. MicroRNA-directed transcriptional gene silencing in mammalian cells. *Proc Natl Acad Sci U S A* 2008; **105**: 16230-16235.

2. Chen L, Yan HX, Yang W, Hu L, Yu LX, Liu Q *et al*. The role of microRNA expression pattern in human intrahepatic cholangiocarcinoma. *J Hepatol* 2009; **50**: 358-369.

3. Noto JM, Piazuelo MB, Chaturvedi R, Bartel CA, Thatcher EJ, Delgado A *et al*. Strain-specific suppression of microRNA-320 by carcinogenic Helicobacter pylori promotes expression of the antiapoptotic protein Mcl-1. *Am J Physiol Gastrointest Liver Physiol* 2013; **305**: G786-796.

4. Schaar DG, Medina DJ, Moore DF, Strair RK, Ting Y. miR-320 targets transferrin receptor 1 (CD71) and inhibits cell proliferation. *Exp Hematol* 2009; **37**: 245-255.

5. Ren XP, Wu J, Wang X, Sartor MA, Qian J, Jones K *et al*. MicroRNA-320 is involved in the regulation of cardiac ischemia/reperfusion injury by targeting heat-shock protein 20. *Circulation* 2009; **119**: 2357-2366.

6. Edwards HV, Cameron RT, Baillie GS. The emerging role of HSP20 as a multifunctional protective agent. *Cell Signal* 2011; **23**: 1447-1454.

7. Saadoun S, Papadopoulos MC, Hara-Chikuma M, Verkman AS. Impairment of angiogenesis and cell migration by targeted aquaporin-1 gene disruption. *Nature* 2005; **434**: 786-792.

8. Sepramaniam S, Armugam A, Lim KY, Karolina DS, Swaminathan P, Tan JR *et al*. MicroRNA 320a functions as a novel endogenous modulator of aquaporins 1 and 4 as well as a potential therapeutic target in cerebral ischemia. *J Biol Chem* 2010; **285**: 29223-29230.

9. Zhang Y, He X, Liu Y, Ye Y, Zhang H, He P *et al*. microRNA-320a inhibits tumor invasion by targeting neuropilin 1 and is associated with liver metastasis in colorectal cancer. *Oncol Rep* 2012; **27**: 685-694.

10. Feng B, Chakrabarti S. miR-320 Regulates Glucose-Induced Gene Expression in Diabetes. *ISRN Endocrinol* 2012; **2012**: 549875.

11. Yao J, Liang LH, Zhang Y, Ding J, Tian Q, Li JJ *et al*. GNAI1 Suppresses Tumor Cell Migration and Invasion and is Post-Transcriptionally Regulated by Mir-320a/c/d in Hepatocellular Carcinoma. *Cancer Biol Med* 2012; **9**: 234-241.

12. Cheng Z, Qiu S, Jiang L, Zhang A, Bao W, Liu P *et al*. MiR-320a is downregulated in patients with myasthenia gravis and modulates inflammatory cytokines production by targeting mitogen-activated protein kinase 1. *J Clin Immunol* 2013; **33**: 567-576.

13. Hsieh IS, Chang KC, Tsai YT, Ke JY, Lu PJ, Lee KH *et al*. MicroRNA-320 suppresses the stem cell-like characteristics of prostate cancer cells by downregulating the Wnt/beta-catenin signaling pathway. *Carcinogenesis* 2013; **34**: 530-538.

14. Ling S, Nanhwan M, Qian J, Kodakandla M, Castillo AC, Thomas B *et al*. Modulation of microRNAs in hypertension-induced arterial remodeling through the beta1 and beta3-adrenoreceptor pathways. *J Mol Cell Cardiol* 2013; **65**: 127-136.

15. Mittal SP, Mathai J, Kulkarni AP, Pal JK, Chattopadhyay S. miR-320a regulates erythroid differentiation through MAR binding protein SMAR1. *Int J Biochem Cell Biol* 2013; **45**: 2519-2529.

16. Cheng C, Chen ZQ, Shi XT. MicroRNA-320 inhibits osteosarcoma cells proliferation by directly targeting fatty acid synthase. *Tumour Biol* 2014; **35**: 4177-4183.

17. He DX, Gu XT, Jiang L, Jin J, Ma X. A methylation-based regulatory network for microRNA 320a in chemoresistant breast cancer. *Mol Pharmacol* 2014; **86**: 536-547.

18. Ou M, Zhang X, Dai Y, Gao J, Zhu M, Yang X *et al*. Identification of potential microRNA-target pairs associated with osteopetrosis by deep sequencing, iTRAQ proteomics and bioinformatics. *Eur J Hum Genet* 2014; **22**: 625-632.

19. Qi X, Li J, Zhou C, Lv C, Tian M. MicroRNA-320a inhibits cell proliferation, migration and invasion by targeting BMI-1 in nasopharyngeal carcinoma. *FEBS Lett* 2014; **588**: 3732-3738.

20. Shang C, Zhang H, Guo Y, Hong Y, Liu Y, Xue Y. MiR-320a down-regulation mediates bladder carcinoma invasion by targeting ITGB3. *Mol Biol Rep* 2014; **41**: 2521-2527.

21. Zhao H, Dong T, Zhou H, Wang L, Huang A, Feng B *et al*. miR-320a suppresses colorectal cancer progression by targeting Rac1. *Carcinogenesis* 2014; **35**: 886-895.

22. Sun JY, Xiao WZ, Wang F, Wang YQ, Zhu YH, Wu YF *et al*. MicroRNA-320 inhibits cell proliferation in glioma by targeting E2F1. *Mol Med Rep* 2015; **12**: 2355-2359.

23. Wan LY, Deng J, Xiang XJ, Zhang L, Yu F, Chen J *et al*. miR-320 enhances the sensitivity of human colon cancer cells to chemoradiotherapy in vitro by targeting FOXM1. *Biochem Biophys Res Commun* 2015; **457**: 125-132.

24. Okato A, Goto Y, Kurozumi A, Kato M, Kojima S, Matsushita R *et al*. Direct regulation of LAMP1 by tumor-suppressive microRNA-320a in prostate cancer. *Int J Oncol* 2016; **49**: 111-122.

25. Xie F, Yuan Y, Xie L, Ran P, Xiang X, Huang Q *et al*. miRNA-320a inhibits tumor proliferation and invasion by targeting c-Myc in human hepatocellular carcinoma. *Onco Targets Ther* 2017; **10**: 885-894.

26. Zhao Y, Dong Q, Wang E. MicroRNA-320 inhibits invasion and induces apoptosis by targeting CRKL and inhibiting ERK and AKT signaling in gastric cancer cells. *Onco Targets Ther* 2017; **10**: 1049-1058.

27. Lv Q, Hu JX, Li YJ, Xie N, Song DD, Zhao W *et al*. MiR-320a effectively suppresses lung adenocarcinoma cell proliferation and metastasis by regulating STAT3 signals. *Cancer Biol Ther* 2017; **18**: 142-151.

28. Iwagami Y, Eguchi H, Nagano H, Akita H, Hama N, Wada H *et al*. miR-320c regulates gemcitabine-resistance in pancreatic cancer via SMARCC1. *Br J Cancer* 2013; **109**: 502-511.

29. Vishnubalaji R, Hamam R, Yue S, Al-Obeed O, Kassem M, Liu FF *et al*. MicroRNA-320 suppresses colorectal cancer by targeting SOX4, FOXM1, and FOXQ1. *Oncotarget* 2016; **7**: 35789-35802.

30. Wang XH, Qian RZ, Zhang W, Chen SF, Jin HM, Hu RM. MicroRNA-320 expression in myocardial microvascular endothelial cells and its relationship with insulin-like growth factor-1 in type 2 diabetic rats. *Clin Exp Pharmacol Physiol* 2009; **36**: 181-188.

31. Bronisz A, Godlewski J, Wallace JA, Merchant AS, Nowicki MO, Mathsyaraja H *et al*. Reprogramming of the tumour microenvironment by stromal PTEN-regulated miR-320. *Nat Cell Biol* 2011; **14**: 159-167.
